# Enriching NPK Mineral Fertilizer with Plant-Stimulating Peptides Increases Soilless Tomato Production, Grower Profit, and Environmental Sustainability

**DOI:** 10.3390/plants13142004

**Published:** 2024-07-22

**Authors:** Michele Ciriello, Sara Rajabi Hamedani, Youssef Rouphael, Giuseppe Colla, Mariateresa Cardarelli

**Affiliations:** 1 Department of Agricultural Sciences, University of Naples Federico II, Via Università 100, 80055 Portici, Italy; michele.ciriello@unina.it (M.C.); youssef.rouphael@unina.it (Y.R.); 2 Department of Agriculture and Forest Sciences, University of Tuscia, Via San Camillo De Lellis Snc, 01100 Viterbo, Italy; sara.rajabi@unitus.it

**Keywords:** biostimulant, hydroponics, life cycle assessment, *Solanum lycopersicum* L., partial budget analysis

## Abstract

The need to increase agricultural production to feed a steadily growing population may clash with the more environmentally friendly but less efficient production methods required. Therefore, it is important to try to reduce the use of chemical inputs without compromising production. In this scenario, natural biostimulants have become one of the most sought-after and researched technologies. In the present study, the results of a greenhouse experiment on hydroponic tomatoes (*Solanum lycopersicum* L.) are presented, which involved comparing the use of ordinary NPK fertilizer (Cerbero^®^) with the use of NPK fertilizers enriched with 0.5% protein hydrolysate of plant origin (Cerbero Green^®^) at both standard (100%) and reduced (70%) fertilization rates. The results highlight how the use of Cerbero Green^®^ fertilizers improves the production performance of tomatoes. More specifically, they show that the use of Cerbero Green^®^ leads to higher marketable yields, especially under reducing fertilizer use, ensuring a positive net change in profit for the grower. In addition, carbon footprint analysis has revealed that the use of Cerbero Green^®^ reduces the environmental impact of hydroponic tomato growing practices by up to 8%. The observed higher yield of hydroponically grown tomatoes even with reduced fertilization rates underlines once again the key role of natural biostimulants in increasing both the economic and environmental sustainability of horticultural production.

## 1. Introduction

Technological progress has driven the agricultural world towards a strong intensification that has, over time, reduced the number of malnourished people, generated employment, and income for farmers [1]. However, the depletion of genetic potential and cultivable areas, coupled with population growth and sudden and unpredictable climate change, has imposed continuous new challenges on the agricultural sector [2,3,4]. One of these is the reduction of energy consumption by improving the efficiency of use of invested resources. Indeed, the reckless application of chemical inputs, such as pesticides and chemical fertilizers, is no longer sustainable due to their serious impact on the environment and human health [5]. Furthermore, it should be noted that some fertilizers used are produced from rock deposits that represent a non-renewable resource [6]. Therefore, the productive agricultural world must ‘move’ towards sustainable development that, by definition, integrates the three dimensions of natural–human systems, namely economic, social, and environmental [5,7,8]. To date, the necessary increase in agricultural production cannot be dissociated from the assessment of nutrient use efficiency (NUE). The excessive use of chemical fertilizers has a cost, both in environmental (ecological footprint, water eutrophication) and economic terms associated with their production, transport, and application [9,10]. For these reasons, it is necessary to maximize NUE to ensure environmental sustainability and economic viability. Specifically, the estimation of NUE is based on two key points as follows: (i) nutrient uptake efficiency, which takes into account the acquisition, inflow, and transport of nutrients into the roots, and (ii) nutrient utilization efficiency, again strongly influenced by the type of crop [11,12]. However, NUE is influenced by a complex and multifaceted set of factors that, in addition to the aforementioned crop characteristics, also takes into account the chemical and physical properties of the soil, climatic parameters, and agronomic management aspects. Therefore, the much-discussed improvement in NUE in plants can only be achieved through careful manipulation of these five key factors. Over the past decades, several technological innovations that have been identified and studied can improve the sustainability of the agricultural world, many of which have been based on increasing NUE [13,14]. The use of biostimulants, which include both natural substances and compounds (e.g., algae extracts, humic acids and protein hydrolysates) as well as beneficial microorganisms (e.g., rhizobacteria and mycorrhizal fungi) are among the most interesting strategies as they ensure that crop yields and quality are improved in a sustainable manner (e.g., by improving NUE) [15]. At the European level, the economic value of the biostimulant industry is estimated at between 200 and 400 million euros (with an average annual growth of around 10%) [16]. Among the various biostimulants, vegetal-derived protein hydrolysates (V-PH) have carved out a prestigious place for themselves in the world of horticulture. In line with the increasingly discussed concepts of circular economy, the production process used to produce these biostimulants (enzymatic hydrolysis) would fit well with agricultural organic waste, transforming it from a problem to be disposed of into a real economic benefit for farmers [16]. Vegetal-derived PHs, besides containing limited amounts of macro- and micro-nutrients, are a rich source of soluble peptides and free amino acids that are mainly responsible for the biostimulating action of these products [17]. Root or foliar applications of plant-derived PHs can trigger the activation of several physiological and molecular mechanisms in different crops, stimulating vegetative growth and resource-use efficiency and consequently improving yield and functional quality [18,19]. For instance, it has been demonstrated that application of the V-PH ‘Trainer^®^’ on greenhouse crops is able to stimulate nutrient uptake and assimilation, with a significant increase in crop productivity [19,20]; this has been linked to the presence of amino acids and small peptides in the biostimulant product, which act as signaling compounds eliciting auxin- and/or gibberellin-like activities on both leaves and roots and thus causing a “nutrient acquisition response” that increases nutrients acquisition and assimilation as well as an increase in the photochemical efficiency and activity of photosystem II [20].

Furthermore, the stimulation of specific protective processes related to osmotic regulation and antioxidant activity provides PH-treated plants with increased ‘protection’ against a wide range of abiotic stresses [21]. Foliar applications of vegetal-derived protein hydrolysate have proven to be able to reduce environmental impact of greenhouse spinach production, as CO_2_ equivalent emissions per unit of spinach yield, especially under reduced nitrogen fertilizer rates [22]. However, there is a lack of information about the effect of vegetal-derived protein hydrolysates on the environmental impact of other important vegetable crops such as greenhouse tomato. Moreover, the biostimulant applications also need to provide appropriate economic profit and competitive advantage for farmers. A previous study on greenhouse tomato demonstrated that foliar applications of vegetal-derived biostimulants enhanced fruit yield, leading to an increase in gross returns that ultimately improved the net returns as compared with the untreated plants [23].

Starting from the above considerations, a greenhouse trial was carried out to evaluate the impact of enriching NPK mineral fertilizers with a V-PH containing plant stimulating peptides on soilless production of greenhouse tomato, environmental indicators, and economic profitability. Environmental indicators were determined only for the first trial using the Life Cycle Assessment, following a cradle-to-gate perspective (plant cultivation phase up to harvest) considering both the direct emissions of the different phases of the process and the indirect emissions associated with the production of raw materials as inputs in the production chain. Moreover, economic profitability, associated with the replacement of NPK fertilizers with NPK fertilizers enriched with plant stimulating peptides, was assessed by partial budget analysis which focuses only on the changes in income and expenses that result from implementing a specific alternative.

## 2. Results

### 2.1. Agronomic Results

The differentiated fertilization management proposed led to significant variations in all yield parameters reported in Table 1. Specifically, for both tested fertilization levels (100% and 70%), the use of NPK fertilizers enriched with 0.5% V-PH (Cerbero Green^®^) resulted in an average increase of 7.3% in fruit yield. A similar trend was partially observed for the number of fruits as well. Indeed, exclusively for plants fertigated at the 100% level, the use of Cerbero Green^®^ compared to Cerbero^®^ resulted in a significant increase (+5%) in this parameter (Table 1). Regarding the average weight of fruits, the use of Cerbero Green^®^ at 70% recorded significantly higher values compared to those obtained with Cerbero Green^®^ at 100%.

### 2.2. LCA Results

The tomato production results varied across different treatments. The highest yield was observed in plants treated with NPK fertilizers enriched with plant stimulating peptides and decreased mineral fertilization (Cerbero Green® 70%). This was followed by plants treated with Cerbero Green® 100%, and plants fertilized with decreased mineral fertilization without biostimulant application (Cerbero® 70%). The lowest fruit yield was obtained from Cerbero® 100%. Considering the functional unit as 1 ton of harvested crop, any increase or decrease in fruit yield had an inverse impact on all environmental indicators.

The findings presented in Table 2 demonstrated that the use of NPK fertilizers containing plant stimulating peptides led to reduced environmental impacts per ton of marketable tomatoes, regardless of whether standard or decreased fertilization was applied. For example, the use of Cerbero Green® resulted in a higher reduction of CO_2_ emissions (−8%) in the plants subjected to decreased fertilization rate compared to those grown under standard fertilization (−5%). Applying protein hydrolysate with standard fertilization treatments resulted in a 4% to 11% decrease in all impact categories, while with decreased fertilization, the reduction ranged from 5% to 9%.

**Figure 1:**
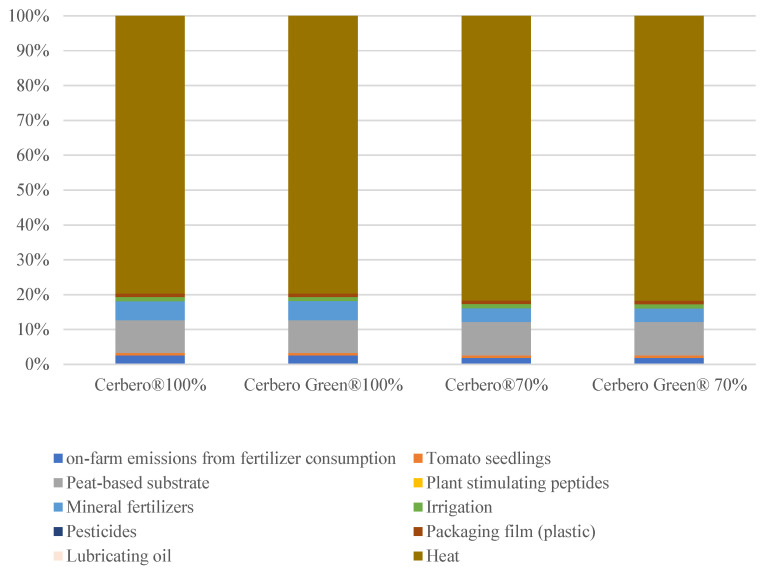
Process contribution to global warming of greenhouse tomato under four treatments per functional unit.

Analyzing the process contributions to the total carbon footprint (Figure 1), it was evident that greenhouse heating due to natural gas consumption was the primary contributor, accounting for 80–82% of the total impact. Other significant environmental burdens were attributed to the production processes of peat-based substrate and mineral fertilizers, as well as on-farm emissions from consumption of these fertilizers, which had substantial impacts on various environmental indicators.

### 2.3. Economic Results

The additional marketable yield resulting from the replacement of Cerbero^®^ with Cerbero Green^®^ was 2.7 and 5.3 t/ha for 100 and 70% level, respectively (Table 1). Therefore, the increases in tomato yield with Cerbero Green^®^ 100% and Cerbero Green^®^ 70% led to an additional gross yield of $2970 and $5830 per hectare, respectively (Table 3). Taking into account the total variable costs associated with the use of Cerbero Green^®^ 100% and Cerbero Green^®^ 70% (Table 3), the net change in profit for plants treated with Cerbero Green^®^ 100% and Cerbero Green^®^ 70% compared to those treated with Cerbero^®^ 100% and Cerbero^®^ 70% was $2365 and $4750 per hectare.

## 3. Discussion

While the literature emphasizes the importance of genotype, environmental conditions, and their interactions on the effects of biostimulants [23,24,25,26], our experiment on tomatoes grown in soilless culture revealed a consistent positive response to biostimulant usage. The application of the Cerbero Green^®^ fertilizers, regardless of the fertilization level (100% and/or 70%), significantly increased the fresh yield of tomatoes, compared to the Cerbero^®^ fertilizers alone. It is noteworthy that the increased production of plants fertigated with Cerbero Green^®^ was primarily attributed to a higher number of fruits per plant, partially confirming what was observed by Rouphael et al. [27]. These results could be attributed to the presence of specific bioactive peptides in Cerbero Green^®^, which have a hormone-like activity in promoting rooting, plant growth, flowering and fruit set. Moreover, vegetal-derived protein hydrolysate (V-PH), besides being characterized by the presence of amino acids and bioactive peptides, may contain traces of other useful compounds such as mineral elements, carbohydrates, phenols, and phytohormones [21]. Although the mode of action of these plant biostimulants is still not completely known today, an increasing amount of data available in the literature highlights the positive effect of V-PH application on the regulation of critical phenological phases such as flowering and fruiting [21,27,28].

The increased number of fruits per plant could be directly related to a better response during the pre-fruiting phase, especially in protected environments where high daytime temperatures can negatively impact this process [27,28]. Additionally, previous studies have shown that V-PH application has a direct impact on root system architecture [29,30]. The improvement in the main parameters related to root architecture (total root area and root length) is directly linked to increased nutrient utilization efficiency and consequently to crop productivity. Specifically, the presence of auxin precursors, root-promoting peptides, and amino acids such as L-glutamate and tryptophan stimulate root growth and the development of absorbing root hairs through specific mechanisms not yet fully understood [27,31]. This improvement in root architecture forms the basis of the efficacy of non-microbial plant biostimulants on nutrient absorption efficiency [32].

The notion that higher fertilization leads to greater production contrasts with current agronomic trends promoting more sustainable practices [33,34]. Excessive fertilizer not only has a negative impact on the environment but also entails high management costs [35,36]. In our study, the use of a lower fertilization level (70%) did not result in reduced yields compared to using Cerbero^®^ at 100%, highlighting the possibility of reducing fertilizer inputs. Furthermore, plants fertigated with Cerbero Green^®^ at 70% showed the highest yields, confirming the effectiveness of biostimulants in reducing mineral input [37,38,39].

Several Life Cycle Assessment (LCA) studies have investigated greenhouse tomato production. Studies indicate the importance of heating systems and energy consumption in global warming potential [40]. Therefore, to facilitate comparisons, the studies have been categorized into heated and unheated greenhouse tomato productions. Table 4 presents a comparison between the LCA results of our current study and several other LCA studies conducted on tomato production in different European countries.

For unheated greenhouse tomato production, Sanjuan-Delmás et al. [41] found that cherry tomato production in an innovative unheated rooftop greenhouse resulted in approximately 580 kg CO_2_ equivalent per 1 ton of tomato. Similarly, Romero-Gámez et al. [42] estimated that tomato production in an unheated greenhouse could lead to approximately 617 kg of CO_2_ per ton of tomato. In our study, excluding heating demand and corresponding impacts, the result ranged from 318 to 367 kg CO_2_ per ton of tomato.

On the other hand, when considering heated greenhouse tomatoes, the outcomes were primarily influenced by the type of fuel used and the amount of energy consumption. Maham et al. [43] examined the environmental performance of greenhouse tomatoes heated by an electric heater under different levels of organic fertilizers and water stress. They estimated an average of 269 kg CO_2_ per ton of tomato. Another study by Bosona and Gebresenbet [44] reported that tomato production in a heated greenhouse using woodchips could entail approximately 547 kg CO_2_ equivalent per ton of crop. However, in our current study, the results were higher, ranging from 1754 to 2029 kg CO_2_ per ton of tomato. This difference can be justified by the application of a natural gas boiler as the heating system in our study.

## 4. Materials and Methods

### 4.1. Plant Material, Treatments, and Experimental Designs

An experiment was conducted in 2021 in a heated polyethylene greenhouse. The average day/night air temperatures were 27.8 ± 1.0/17.3 ± 0.9 °C. Tomato plants (*Solanum lycopersicum* L.) were grown in bags filled with 100% coconut fiber (Planet Agro, Créon, France); each bag contained 3 plants, providing a planting density of 2 pt/m^2^. Tomato plants of the cultivars Kalixo HF1 (Gautier Semences; Eyragues, Arles, France) were transplanted at the three-true leaf stage on April 11. Randomized complete block design with four replicates was used. Two levels of fertilization (100% and 70%) were examined, using conventional water-soluble NPK fertilizers (hereinafter referred to as Cerbero^®^) and NPK fertilizers enriched with 0.5% vegetal-derived protein hydrolysate (V-PH) containing plant stimulating peptides (hereinafter referred to as Cerbero Green^®^). The V-PH contained 75% of organic compounds as peptides and amino acids, resulting from enzymatic hydrolysis of legume seeds. The aminogram was as follows: 4.6% Alanine, 7.0% Arginine, 11.7% Aspartic acid, 1.0% Cysteine, 18.0% Glutamic acid, 4.5% Glycine, 2.8% Histidine, 4.8% Isoleucine, 8.0% Leucine, 6.0% Lysine, 1.5% Methionine, 5.2% Phenylalanine, 5.1% Proline, 5.5% Serine, 4.1% Threonine, 1.2% Tryptophan, 3.9% Tyrosine, 5.1% Valin. Moreover, V-PH contains 22% of soluble carbohydrates and 3% of mineral elements. The fertilization plan was set up according to the commercial fertilizer software GSC06 developed by Greenspec Company (Groningen; The Netherlands—www.greenspec.nl).

Overall, four treatments were implemented, each replicated four times, with five plants per replication. The complete fertilization plan is detailed in Table 5. Both NPK fertilizers (Cerbero^®^ and Cerbero Green^®^) were manufactured by Hello Nature Inc. (Anderson, IN, USA).

Fertigation was performed using a drip irrigation system having one emitter per plant of 2 L/h. Fertigation was managed to assure that at least 30% of the drainage from the bags to avoid salt build up into the substrate. Pests and diseases were controlled by commercial pesticides at the labelled rates.

### 4.2. Inventory Data Collection

The experiment lasted 169 days (from 11 April to 27 September). At each harvest, the fruits from each treatment were counted, weighed, and separated into two groups, namely non-marketable fruits (green and/or deformed) and marketable fruits (free of visible defects and mature), to determine the marketable fruit yield, fruit number, and fruit mean weight. Moreover, all inputs, like peat-based substrate, mineral fertilizers, protein hydrolysate, irrigation water, pesticides, plastic, lubricant, heating, used in the cropping cycle were recorded and used for calculation of environmental indicators and carbon foot printing.

The necessary data for modeling the greenhouse tomato product system, particularly foreground data, were obtained from an experimental farm affiliated with Tuscia University. These foreground data encompassed critical information such as fertilizer quantities, seedling numbers, pesticide usage, and water consumption pertinent to tomato cultivation.

In contrast, background data concerning the production of input materials such as energy, seeds, and mineral fertilizers were sourced from the Ecoinvent database. This dataset contributes to offering a comprehensive understanding of the environmental impacts associated with the entire life cycle of greenhouse tomato production. In the case of V-PH production, the presentation of energy and material balances was omitted due to agreements regarding confidential data disclosure. Additionally, emissions occurring on-farm, notably those arising from fertilizer application, were computed utilizing the data outlined in Table 6 and Table 7.

Subsequently, the outcomes of these computations were consolidated and presented in Table 8. Furthermore, the inventory encompasses the process of biostimulant production (plant stimulating peptides), specifically sourced from soybeans.

### 4.3. Life Cycle Assessment and Carbon Footprint

Life Cycle Assessment and Carbon Footprint was applied only to greenhouse tomato production.

The LCA model was developed in SimaPro software (version 9.5.0.0). In this study, the ReCiPe 2016 midpoint v1.03 method converted the data inventory into conversational indicators [40]. In order to comprehend the significance of indicators, the environmental results were normalized. Therefore, among a total of eighteen impact categories assessed, the following eleven categories with significant effects have been identified: Global warming, Ozone formation—human health, Ozone formation—terrestrial ecosystems, Freshwater eutrophication, Terrestrial ecotoxicity, Freshwater ecotoxicity, Marine ecotoxicity, Human carcinogenic toxicity, Human non-carcinogenic toxicity, Fossil resource scarcity, and Water consumption.

Global warming, as measured using the IPCC methodology, has been assessed over a span of 100 years. The concept of global warming potential quantifies the extra radiative forces accumulated over a century due to the emission of 1 kg of greenhouse gas compared to the emission of the same mass of CO_2_ over the same period. A large set of greenhouse gas emissions (207 GHGs in total) is involved in measuring global warming potential. This comprises a range of gases such as carbon dioxide, methane, nitrogen oxide, chlorofuorocarbons, hydrochlorofluoro carbons, hydrofluorocarbons, chlorocarbons and hydrochlorocarbons, bromocarbons, hyrdobromocarbons and halons, fully fluorinated species and halogenated alcohols and ethers. The global warming potentials (kg CO_2_ eq per kg greenhouse gases) are presented in Table A1 in Appendix A.

### 4.4. Partial Budget Analysis

In line with the procedure previously described by Djidonou et al. [46], a partial budget analysis was performed to assess the cost-effectiveness of replacing standard NPK fertilizers (Cerbero^®^) with the NPK fertilizers enriched with plant stimulating peptides (Cerbero Green^®^). Compared to control conditions (fertilization program based on the use of Cerbero^®^), the gross added yield and added costs of using the fertilization program with Cerbero Green^®^ were calculated and, from these, the net added yield was calculated by difference.

### 4.5. Statistical Analysis

All data were subjected to ANOVA using the SPSS22 software package (Chicago, IL, USA). Means were separated using Tukey’s range test performed at 5% level of significance.

## 5. Conclusions

The growing need to increase agricultural production to support a continuously expanding population has prompted the scientific community to propose alternative and sustainable production technologies. In this context, biostimulants have played and are playing a key role. In addition to reducing the incidence of abiotic stress, an increasing number of studies have begun to assess the possibility of reducing the use of chemical inputs such as mineral fertilizers by using natural products like biostimulants. The results of our experiment confirm how the use of NPK fertilizers enriched with V-PH (Cerbero Green^®^) improves the productive performance of soilless-grown tomatoes. Specifically, the results have shown that the use of Cerbero Green^®^ leads to higher marketable yields while reducing fertilizer usage and simultaneously ensuring a positive net change in profit for the grower. In addition, the carbon footprint estimation results revealed that using Cerbero Green^®^ could reduce the global warming potential of greenhouse-grown tomatoes by 5–8%. This positive outcome was primarily attributed to the increased productivity of the crops. Additionally, when considering other impact categories, the use of Cerbero Green^®^ demonstrated reductions of 4–11% and 5–9% in standard and decreased fertilization scenarios, respectively. These findings offer valuable insights into the sustainable management of vegetable crops, especially regarding the effective utilization of vegetal-derived protein hydrolysates containing plant stimulating peptides as additives of mineral fertilizers.

## Figures and Tables

**Table 1:** Effect of fertilization type and rate on marketable fruit yield and yield components of soilless tomato.

Treatment	Fruit Yield (t/ha)	Fruit Number (n/m^2^)	Fruit Mean Weight (g/fruit)
Cerbero^®^ 100%	53.9 ± 0.3 c	39.1 ± 0.1 b	138.5 ± 1.5 ab
Cerbero Green^®^ 100%	56.6 ± 0.2 b	42.1 ± 0.1 a	134.2 ± 0.1 b
Cerbero^®^ 70%	55.5 ± 0.6 bc	40.0 ± 1.3 ab	138.7 ± 1.7 ab
Cerbero Green^®^ 70%	60.8 ± 0.6 a	42.4 ± 0.2 a	143.5 ± 1.8 a
Significance	***	**	*

Data are the average of four replicates ± standard error. *, **, *** means significant at *p* ≤ 0.05, 0.01, 0.001, respectively. The different letters indicate significant difference according to the Tukey’s range test, *p* ≤ 0.05.

**Table 2:** Comparative environmental results of greenhouse tomato under different fertilizer treatments (ReCipe 2016, FU: 1 t).

Impact Category	Unit	Cerbero^®^ 100%	Cerbero Green^®^ 100%	Cerbero^®^ 70%	Cerbero Green^®^ 70%
Global warming	kg CO_2_ eq	2029.51	1932.96	1923.08	1753.95
Ozone formation, Human health	kg NOx eq	1.28	1.22	1.18	1.08
Ozone formation, Terrestrial ecosystems	kg NOx eq	1.33	1.27	1.23	1.12
Freshwater eutrophication	kg P eq	0.05	0.05	0.04	0.04
Terrestrial ecotoxicity	kg 1,4-DCB	472.65	450.37	371.72	353.31
Freshwater ecotoxicity	kg 1,4-DCB	4.68	4.46	3.89	3.65
Marine ecotoxicity	kg 1,4-DCB	9.48	9.04	8.27	7.69
Human carcinogenic toxicity	kg 1,4-DCB	9.13	8.70	8.31	7.59
Human non-carcinogenic toxicity	kg 1,4-DCB	133.35	127.16	108.67	103.31
Fossil resource scarcity	kg oil eq	722.56	688.16	696.49	635.63
Water consumption	m^3^	69.36	66.06	66.90	60.74

**Table 3:** Additional revenue and variable costs, and net change in grower profit resulting from the replacement of Cerbero^®^ by Cerbero Green^®^ fertilizer at the same fertilization rate.

Treatment	Additional Revenue ($/ha)	Additional Variable Cost ($/ha)	Net Change in Profit ($/ha)
Cerbero Green^®^ 100%	2970	605	2365
Cerbero Green^®^ 70%	5830	1080	4750

The tomato selling price of 1.10 $/kg was used in the calculation of gross margin. The added variable costs include the additional cost resulting from the extra cost of replacing Cerbero^®^ with Cerbero Green^®^ fertilizers and the additional cost for harvesting the supplemental yield gained with Cerbero Green^®^ application instead of regular Cerbero^®^.

**Table 4:** Comparison of greenhouse gas emission of the current study with some existing studies in other European countries.

System Description	Impact Category	Quantity (kg CO_2_ per 1 Ton of Tomato)	Reference
Greenhouse tomato with biostimulant application heated by natural gas; scope of grate to gate	GWP	1754–2029	Current study
Unheated organic greenhouse tomato in Spain; scope of cradle to consumer gate	GWP	580	[41]
Unheated conventional greenhouse tomato in Spain; scope of cradle to farm gate	GWP	617	[42]
Organic greenhouse tomato heated by electric heater in Canada; scope of cradle to farm gate	GWP	269	[43]
Organic greenhouse tomato heated by woodchips in Sweden; scope of cradle to consumer gate	GWP	547	[44]

GWP = Global Warming Potential.

**Table 5:** Full fertilization plan (named 100%) used in the tomato production.

Fertilizer Type	Fertilizer Rate (g/L)						
	<2nd Cluster	3rd Cluster	4th Cluster	5th Cluster	6th Cluster	7th Cluster	>8th Cluster
Cerbero^®^ or Cerbero Green^®^ (13% N; 40% P_2_O_5_; 13% K_2_O; 2% MgO)	0.50	0.80	0.00	0.00	0.00	0.00	0.00
Cerbero^®^ or Cerbero Green^®^ (15% N; 5% P_2_O_5_; 30% K_2_O; 2% MgO)	0.00	0.00	1.00	1.00	1.00	1.10	1.10
Potassium nitrate (13.5% N; 46.2% K_2_O)	0.00	0.00	0.50	0.50	0.30	0.30	0.30
Magnesium nitrate (11% N; 16% MgO)	0.25	0.25	0.25	0.25	0.50	0.50	0.50
Calcium nitrate (15.5% N; 26.5% CaO)	0.25	0.25	0.25	0.25	1.20	1.15	1.15
Iron chelate (6% Fe-EDDHA)	0.025	0.025	0.025	0.025	0.025	0.025	0.05
Microelement complex (4%Fe; 4% Mn; 1% Zn; 0.5% Cu; 0.5% B; 0.2% Mo)	0.025	0.025	0.025	0.025	0.025	0.025	0.025

Cerbero^®^ contained mineral nutrients while Cerbero Green^®^ contained mineral nutrients enriched with 0.5% vegetal-derived protein hydrolysate.

**Table 6:** Coefficients for conversion of emissions.

Emissions	Coefficient
kg N_2_O-N to kg N_2_O	[28,44]

**Table 7:** Coefficients for calculating on-farm emissions related to the application of fertilisers for tomato production.

Characteristics	Coefficient	Emission Fate
Emissions from mineral fertilizers		[45]
N in mineral fertilizer	0.01	N_2_O-N to air
Indirect N_2_O from atmospheric deposition of fertilizers		[45]
N in mineral fertilizer	0.01 × 0.1	N_2_O-N to air

**Table 8:** Inventory data for tomato production in relation to fertilizer treatments.

Items	Unit	Quantity (Unit ha^−1^)			
		Cerbero^®^ 100%	Cerbero Green^®^ 100%	Cerbero^®^ 70%	Cerbero Green^®^ 70%
Output to technosphere					
Fruit yield	t	53.9	56.6	55.5	60.8
Input from technosphere					
Seedlings	n	20,000	20,000	20,000	20,000
Peat-based substrate	m^3^	80	80	80	80
Mineral fertilizers	kg	1515	1515	1061	1061
Vegetal-derived protein hydrolysate	kg	0	8.60	0	8.60
Irrigation	m^3^	3600	3600	3600	3600
Deltamethrin	g	75	75	75	75
Abamectin	g	40	40	40	40
Copper oxychloride	kg	3	3	3	3
Plastic	kg	360	360	360	360
Lubricant	kg	5	5	5	5
Heating (natural gas)	GJ	1259	1259	1259	1259
Output to environment					
Emission to air					
N_2_O	kg	8.63	8.63	6.03	6.03
Indirect N_2_O	kg	0.86	0.86	0.60	0.60

## Data Availability

Data supporting reported results are available upon request to the corresponding authors.

## Appendix A

**Table A1:** Global Warming Potential (kg CO_2_ eq/kg greenhouse gas) over a span of 100 years [1].

Name	Formula	Hierarchist (100 Years)
Carbon dioxide	CO_2_	1
Methane	CH_4_	34
Fossil methane	CH_4_	36
Nitrous oxide	N_2_O	298
**Chlorofluorocarbons**		
CFC-11	CCl3F	5352
CFC-12	CCl2F2	11,547
CFC-13	CClF3	15,451
CFC-113	CCl2FCClF2	6586
CFC-114	CClF2CClF2	9615
CFC-115	CClF2CF3	8516
**Hydrochlorofluoro-carbons**		
HCFC-21	CHCl2F	179
HCFC-22	CHClF2	2106
HCFC-122	CHCl2CF2Cl	72
HCFC-122a	CHFClCFCl2	312
HCFC-123	CHCl2CF3	96
HCFC-123a	CHClFCF2Cl	447
HCFC-124	CHClFCF3	635
HCFC-132c	CH2FCFCl2	409
HCFC-141b	CH3CCl2F	938
HCFC-142b	CH3CClF2	2345
HCFC-225ca	CHCl2CF2CF3	155
HCFC-225cb	CHClFCF2CClF2	633
(E)-1-Chloro-3,3,3- trifluoroprop-1-ene	trans- CF3CH=CHC1	2
**Hydrofluorocarbons**		
HFC-23	CHF3	13,856
HFC-32	CH2F2	817
HFC-41	CH3F	141
HFC-125	CHF2CF3	3691
HFC-134	CHF2CHF2	1337
HFC-134a	CH2FCF3	1549
HFC-143	CH2FCHF2	397
HFC-143a	CH3CF3	5508
HFC-152	CH2FCH2F	20
HFC-152a	CH3CHF2	167
HFC-161	CH3CH2F	4
HFC-227ca	CF3CF2CHF2	3077
HFC-227ea	CF3CHFCF3	3860
HFC-236cb	CH2FCF2CF3	1438
HFC-236ea	CHF2CHFCF3	1596
HFC-236fa	CF3CH2CF3	8998
HFC-245ca	CH2FCF2CHF2	863
HFC-245cb	CF3CF2CH3	5298
HFC-245ea	CHF2CHFCHF2	285
HFC-245eb	CH2FCHFCF3	352
HFC-245fa	CHF2CH2CF3	1032
HFC-263fb	CH3CH2CF3	92
HFC-272ca	CH3CF2CH3	175
HFC-329p	CHF2CF2CF2CF3	2742
HFC-365mfc	CH3CF2CH2CF3	966
HFC-43-10mee	CF3CHFCHFCF2C F3	1952
HFC-1132a	CH2=CF2	0
HFC-1141	CH2=CHF	0
(Z)-HFC-1225ye	CF3CF=CHF(Z)	0
(E)-HFC-1225ye	CF3CF=CHF(E)	0
(Z)-HFC-1234ze	CF3CH=CHF(Z)	0
HFC-1234yf	CF3CF=CH2	0
(E)-HFC-1234ze	trans- CF3CH=CHF	1
(Z)-HFC-1336	CF3CH=CHCF3(Z)	2
HFC-1243zf	CF3CH=CH2	0
HFC-1345zfc	C2F5CH=CH2	0
3,3,4,4,5,5,6,6,6-	C4F9CH=CH2	0
Nonafluorohex-1-ene		
3,3,4,4,5,5,6,6,7,7,8,8,8	C6F13CH=CH2	0
-Tridecafluorooct-1-ene		
3,3,4,4,5,5,6,6,7,7,8,8,9	C8F17CH=CH2	0
,9,10,10,10-		
Heptadecafluorodecene		
**Chlorocarbons and hydrochlorocarbons**		
Methyl chloroform	CH3CCl3	193
Carbon tetrachloride	CCl4	2019
Methyl chloride	CH3Cl	15
Methylene chloride	CH2Cl2	11
Chloroform	CHCl3	20
1,2-Dichloroethane	CH2ClCH2Cl	1
**Bromocarbons, hyrdobromocarbons and Halons**		
Methyl bromide	CH3Br	3
Methylene bromide	CH2Br2	1
Halon-1201	CHBrF2	454
Halon-1202	CBr2F2	280
Halon-1211	CBrClF2	2070
Halon-1301	CBrF3	7154
Halon-2301	CH2BrCF3	210
Halon-2311/Halothane	CHBrClCF3	50
Halon-2401	CHFBrCF3	223
Halon-2402	CBrF2CBrF2	1734
**Fully Fluorinated Species**		
Nitrogen trifluoride	NF3	17,885
Sulphur hexafluoride	SF6	26,087
(Trifluoromethyl) sulfur	SF5CF3	19,396
pentafluoride		
Sulfuryl fluoride	SO2F2	4732
PFC-14	CF4	7349
PFC-116	C2F6	12,340
PFC-c216	c-C3F6	10,208
PFC-218	C3F8	9878
PFC-318	c-C4F8	10,592
PFC-31-10	C4F10	10,213
Perfluorocyclopentene	c-C5F8	2
PFC-41-12	n-C5F12	9484
PFC-51-14	n-C6F14	8780
PFC-61-16	n-C7F16	8681
PFC-71-18	C8F18	8456
PFC-91-18	C10F18	7977
Perfluorodecalin(cis)	Z-C10F18	8033
Perfluorodecalin(trans)	E-C10F18	6980
PFC-1114	CF2=CF2	0
PFC-1216	CF3CF=CF2	0
Perfluorobuta-1,3-diene	CF2=CFCF=CF2	0
Perfluorobut-1-ene	CF3CF2CF=CF2	0
Perfluorobut-2-ene	CF3CF=CFCF3	2
**Halogenated alcohols and ethers**		
HFE-125	CHF2OCF3	13,951
HFE-134 (HG-00)	CHF2OCHF2	6512
HFE-143a	CH3OCF3	632
HFE-227ea	CF3CHFOCF3	7377
HCFE-235ca2(enflurane)	CHF2OCF2CHFCl	705
HCFE-235da2(isoflurane)	CHF2OCHClCF3	595
HFE-236ca	CHF2OCF2CHF2	4990
HFE-236ea2(desflurane)	CHF2OCHFCF3	2143
HFE-236fa	CF3CH2OCF3	1177
HFE-245cb2	CF3CF2OCH3	790
HFE-245fa1	CHF2CH2OCF3	997
HFE-245fa2	CHF2OCH2CF3	981
2,2,3,3,3-	CF3CF2CH2OH	23
Pentafluoropropane-1-ol		
HFE-254cb1	CH3OCF2CHF2	365
HFE-263fb2	CF3CH2OCH3	2
HFE-263m1	CF3OCH2CH3	26
3,3,3-Trifluoropropan-1- ol	CF3CH2CH2OH	0
HFE-329mcc2	CHF2CF2OCF2CF 3	3598
HFE-338mmz1	(CF3)2CHOCHF2	3081
HFE-338mcf2	CF3CH2OCF2CF3	1118
Sevoflurane (HFE-	(CF3)2CHOCH2F	262
347mmz1)		
HFE-347mcc3 (HFE-	CH3OCF2CF2CF3	641
7000)		
HFE-347mcf2	CHF2CH2OCF2CF3	1028
HFE-347pcf2	CHF2CF2OCH2CF3	1072
HFE-347mmy1	(CF3)2CFOCH3	440
HFE-356mec3	CH3OCF2CHFCF3	468
HFE-356mff2	CF3CH2OCH2CF3	20
HFE-356pcf2	CHF2CH2OCF2C HF2	867
HFE-356pcf3	CHF2OCH2CF2C	540
HFE-356pcc3	CH3OCF2CF2CHF2	500
HFE-356mmz1	(CF3)2CHOCH3	17
HFE-365mcf3	CF3CF2CH2OCH3	1
HFE-365mcf2	CF3CF2OCH2CH3	71
HFE-374pc2	CHF2CF2OCH2CH3	758
4,4,4-Trifluorobutan-1-ol	CF3(CH2)2CH2OH	0
2,2,3,3,4,4,5,5-	(CF2)4CH(OH)	16
Octafluorocyclopentanol		
HFE-43-10pccc124(H-	CHF2OCF2OC2F4	3353
Galden 1040x,HG-11)	OCHF2	
HFE-449s1 (HFE-7100)	C4F9OCH3	509
n-HFE-7100	n-C4F9OCH3	587
i-HFE-7100	i-C4F9OCH3	492
HFE-569sf2 (HFE-7200)	C4F9OC2H5	69
n-HFE-7200	n-C4F9OC2H5	79
i-HFE-7200	i-C4F9OC2H5	54
HFE-236ca12 (HG-10)	CHF2OCF2OCHF2	6260
HFE-338pcc13 (HG-01)	CHF2OCF2CF2OCHF2	3466
1,1,1,3,3,3-	(CF3)2CHOH	221
Hexafluoropropane-2-ol		
HG-02	HF2C–(OCF2CF2)2– OCF2H	3250
HG-03	HF2C–(OCF2CF2)3– OCF2H	3400
HG-20	HF2C–(OCF2)2– OCF2H	6201
HG-21	HF2C– OCF2CF2OCF2OC F2O–CF2H	4628
HG-30	HF2C–(OCF2)3– OCF2H	8575
1-Ethoxy-1,1,2,2,3,3,3-	CF3CF2CF2OCH2 CH3	74
heptafluoropropane		
Fluoroxene	CF3CH2OCH=CH2	0
1,1,2,2-Tetrafluoro-1-	CH2FOCF2CF2H	1051
(fluoromethoxy)ethane		
2-Ethoxy-3,3,4,4,5-	C12H5F19O2	68
pentafluorotetrahydro-		
2,5-bis[1,2,2,2-		
tetrafluoro-1-		
(trifluoromethyl)ethyl]-		
furan		
Fluoro(methoxy)methane	CH3OCH2F	15
Difluoro(methoxy)methane	CH3OCHF2	175
Fluoro(fluoromethoxy)-	CH2FOCH2F	159
methane		
Difluoro(fluoromethoxy)-	CH2FOCHF2	748
methane		
Trifluoro(fluoromethoxy)-	CH2FOCF3	909
methane		
HG'-01	CH3OCF2CF2OC H3	269
HG'-02	CH3O(CF2CF2O) 2CH3	287
HG'-03	CH3O(CF2CF2O) 3CH3	268
HFE-329me3	CF3CFHCF2OCF3	5241
3,3,4,4,5,5,6,6,7,7,7-	CF3(CF2)4CH2C H2OH	1
Undecafluoroheptan-1-ol		
3,3,4,4,5,5,6,6,7,7,8,8,9	CF3(CF2)6CH2C H2OH	0
,9,9- Pentadecafluorononan-1-ol		
		
3,3,4,4,5,5,6,6,7,7,8,8,9	CF3(CF2)8CH2C H2OH	0
,9,10,10,11,11,11-		
Nonadecafluoroundecan-1-ol		
2-Chloro-1,1,2-trifluoro-	CH3OCF2CHFCl	149
1-methoxyethane		
PFPMIE(perfluoropoly- methylisopropyl ether)	CF3OCF(CF3)CF2 OCF2OCF3	10,789
HFE-216	CF3OCF=CF2	0
Trifluoromethylformate	HCOOCF3	712
Perfluoroethylformate	HCOOCF2CF3	703
Perfluoropropylformate	HCOOCF2CF2CF3	456
Perfluorobutylformate	HCOOCF2CF2CF2 CF3	475
2,2,2- Trifluoroethylformate	HCOOCH2CH2CF3	41
3,3,3- Trifluoropropylformate	HCOOCHFCF3	21
1,2,2,2- Tetrafluoroethylformate	HCOOCHFCF3	569
1,1,1,3,3,3- Hexafluoropropan-2- ylformate	HCOOCH(CF3)2	403
Perfluoropropylacetate	CH3COOCF2CF2 CF2CF3	2
Perfluoroethylacetate	CH3COOCF2CF2 CF3	2
Perfluorobutylacetate	CH3COOCF2CF3	3
Trifluoromethylacetate	CH3COOCF3	3
Methylcarbonofluoridate	FCOOCH3	116
1,1- Difluoroethylcarbonofluoridate	FCOOCF2CH3	33
1,1-Difluoroethyl2,2,2- trifluoroacetate	CF3COOCH2CF3	38
Ethyl 2,2,2- trifluoroacetate	CF3COOCH2CH3	2
2,2,2-Trifluoroethyl2,2,2-trifluoroacetate	CF3COOCH2CF3	8
Methyl 2,2,2- trifluoroacetate	CF3COOCH3	64
Methyl 2,2-difluoroacetate	HCF2COOCH3	4
Difluoromethyl 2,2,2-	CF3COOCHF2	33
trifluoroacetate 2,2,3,3,4,4,4- Heptafluorobutan-1-ol	C3F7CH2OH	41
1,1,2-Trifluoro-2- (trifluoromethoxy)- ethane	CHF2CHFOCF3	1489
1-Ethoxy-1,1,2,3,3,3-	CF3CHFCF2OCH2 CH3	28
hexafluoropropane		
1,1,1,2,2,3,3- Heptafluoro-3-(1,2,2,2- tetrafluoroethoxy)- propane	CF3CF2CF2OCHF CF3	7371
2,2,3,3-Tetrafluoro- 1- propanol	CHF2CF2CH2OH	16
2,2,3,4,4,4-Hexafluoro- 1-butanol	CF3CHFCF2CH2OH	21
2,2,3,3,4,4,4- Heptafluoro-1-butanol	CF3CF2CF2CH2OH	20
1,1,2,2-Tetrafluoro-3- methoxy-propane	CHF2CF2CH2OC H3	1
perfluoro-2-methyl-3- pentanone	CF3CF2C(O)CF(C F3)2	0
3,3,3-Trifluoropropanal	CF3CH2CHO	0
2-Fluoroethanol	CH2FCH2OH	1
2,2-Difluoroethanol	CHF2CH2OH	4
2,2,2-Trifluoroethanol	CF3CH2OH	24
1,1’-Oxybis[2- (difluoromethoxy)- 1,1,2,2-tetrafluoroethane	HCF2O(CF2CF2O)2CF2H	5741
1,1,3,3,4,4,6,6,7,7,9,9,10,10,12,12- hexadecafluoro-2,5,8,11- Tetraoxadodecane	HCF2O(CF2CF2O)3CF2H	5245
1,1,3,3,4,4,6,6,7,7,9,9,10,10,12,12,13,13,15,15- eicosafluoro-2,5,8,11,14-Pentaoxapentadecane	HCF2O(CF2CF2O)4CF2H	4240

## References

[1] Szparaga A., Kuboń M., Kocira S., Czerwińska E., Pawłowska A., Hara P., Kobus Z., Kwaśniewski D. (2019). Towards sustainable agriculture—Agronomic and economic effects of biostimulant use in common bean cultivation. Sustainability.

[2] Fan S., Pandya-Lorch R., Yosef S. (2014). Resilience for Food and Nutrition Security.

[3] Food and Agriculture Organization of the United Nations (2012). Towards the Future We Want: End Hunger and Make the Transition to Sustainable Agricultural and Food Systems.

[4] Shahzad A., Ullah S., Dar A.A., Sardar M.F., Mehmood T., Tufail M.A., Shakoor A., Haris M. (2021). Nexus on climate change: Agriculture and possible solution to cope future climate change stresses. Environ. Sci. Pollut. Res..

[5] Aznar-Sánchez J.A., Velasco-Muñoz J.F., López-Felices B., Román-Sánchez I.M. (2020). An analysis of global research trends on greenhouse technology: Towards a sustainable agriculture. Int. J. Environ. Res. Public Health.

[6] Nieves-Cordones M., Rubio F., Santa-María G.E. (2020). Nutrient use-efficiency in plants: An integrative approach. Front. Plant Sci..

[7] Hong C., Burney J.A., Pongratz J., Nabel J.E., Mueller N.D., Jackson R.B., Davis S.J. (2021). Global and regional drivers of land-use emissions in 1961–2017. Nature.

[8] Viana C.M., Freire D., Abrantes P., Rocha J., Pereira P. (2022). Agricultural land systems importance for supporting food security and sustainable development goals: A systematic review. Sci. Total Environ..

[9] Baum R., Bieńkowski J. (2020). Eco-efficiency in measuring the sustainable production of agricultural crops. Sustainability.

[10] Martínez-Dalmau J., Berbel J., Ordóñez-Fernández R. (2021). Nitrogen fertilization. A review of the risks associated with the inefficiency of its use and policy responses. Sustainability.

[11] Mathur M., Goel A. (2017). Quantitative attributes of nutrient uptake and use efficiency. Essential Plant Nutrients.

[12] Reich M., Aghajanzadeh T., De Kok L.J. (2014). Physiological basis of plant nutrient use efficiency–concepts, opportunities and challenges for its improvement. Nutrient Use Efficiency in Plants.

[13] Khan N., Ray R.L., Sargani G.R., Ihtisham M., Khayyam M., Ismail S. (2021). Current progress and future prospects of agriculture technology: Gateway to sustainable agriculture. Sustainability.

[14] Ruzzante S., Labarta R., Bilton A. (2021). Adoption of agricultural technology in the developing world: A meta-analysis of the empirical literature. World Dev..

[15] Calvo P., Nelson L., Kloepper J.W. (2014). Agricultural uses of plant biostimulants. Plant Soil.

[16] Xu L., Geelen D. (2018). Developing biostimulants from agro-food and industrial by-products. Front. Plant Sci..

[17] Meddich A. (2023). Biostimulants for resilient agriculture—Improving plant tolerance to abiotic stress: A concise review. Gesunde Pflanz..

[18] Baltazar M., Correia S., Guinan K.J., Sujeeth N., Bragança R., Gonçalves B. (2021). Recent advances in the molecular effects of biostimulants in plants: An overview. Biomolecules.

[19] Sestili F., Rouphael Y., Cardarelli M., Pucci A., Bonini P., Canaguier R., Colla G. (2018). Protein hydrolysate stimulates growth in tomato coupled with N-dependent gene expression involved in N assimilation. Front. Plant Sci..

[20] Carillo P., Colla G., Fusco G.M., Dell’Aversana E., El-Nakhel C., Giordano M., Pannico A., Cozzolino E., Mori M., Reynaud H. (2019). Morphological and Physiological Responses Induced by Protein Hydrolysate-Based Biostimulant and Nitrogen Rates in Greenhouse Spinach. Agronomy.

[21] Malécange M., Sergheraert R., Teulat B., Mounier E., Lothier J., Sakr S. (2023). Biostimulant Properties of Protein Hydrolysates: Recent Advances and Future Challenges. Int. J. Mol. Sci..

[22] Rajabi Hamedani S., Rouphael Y., Colla G., Colantoni A., Cardarelli M. (2020). Biostimulants as a tool for improving environmental sustainability of greenhouse vegetable crops. Sustainability.

[23] Colla G., Cardarelli M., Bonini P., Rouphael Y. (2017). Foliar applications of protein hydrolysate, plant and seaweed extracts increase yield but differentially modulate fruit quality of greenhouse tomato. Hortscience.

[24] Franzoni G., Bulgari R., Florio F.E., Gozio E., Villa D., Cocetta G., Ferrante A. (2023). Effect of biostimulant raw materials on soybean (*Glycine max*) crop, when applied alone or in combination with herbicides. Front. Agron..

[25] Gómez E., Alonso A., Sánchez J., Muñoz P., Marín J., Mostaza-Colado D., Mauri P.V. (2024). Application of Biostimulant in Seeds and Soil on Three Chickpea Varieties: Impacts on Germination, Vegetative Development, and Bacterial Facilitation of Nitrogen and Phosphorus. Life.

[26] Yakhin O.I., Lubyanov A.A., Yakhin I.A., Brown P.H. (2017). Biostimulants in plant science: A global perspective. Front. Plant Sci..

[27] Rouphael Y., Colla G., Giordano M., El-Nakhel C., Kyriacou M.C., De Pascale S. (2017). Foliar applications of a legume-derived protein hydrolysate elicit dose-dependent increases of growth, leaf mineral composition, yield and fruit quality in two greenhouse tomato cultivars. Sci. Hortic..

[28] Parađiković N., Teklić T., Zeljković S., Lisjak M., Špoljarević M. (2019). Biostimulants research in some horticultural plant species—A review. Food Energy Secur..

[29] Ceccarelli A.V., Miras-Moreno B., Buffagni V., Senizza B., Pii Y., Cardarelli M., Rouphael Y., Colla G., Lucini L. (2021). Foliar application of different vegetal-derived protein hydrolysates distinctively modulates tomato root development and metabolism. Plants.

[30] Santi C., Zamboni A., Varanini Z., Pandolfini T. (2017). Growth stimulatory effects and genome-wide transcriptional changes produced by protein hydrolysates in maize seedlings. Front. Plant Sci..

[31] Halpern M., Bar-Tal A., Ofek M., Minz D., Muller T., Yermiyahu U. (2015). The use of biostimulants for enhancing nutrient uptake. Adv. Agron..

[32] Rouphael Y., Colla G. (2020). Toward a sustainable agriculture through plant biostimulants: From experimental data to practical applications. Agronomy.

[33] Bathaei A., Štreimikienė D. (2023). A Systematic Review of Agricultural Sustainability Indicators. Agriculture.

[34] Lankoski J., Lankoski L. (2023). Environmental sustainability in agriculture: Identification of bottlenecks. Ecol. Econ..

[35] Achakzai A.G., Gul S., Buriro A.H., Khan H., Mushtaq A., Bano A., Agha S., Kamran K., Ponya Z., Ismail T. (2023). Biochar-fertilizer mixture: Does plant life history trait determine fertilizer application rate?. Environ. Pollut. Bioavailab..

[36] Penuelas J., Coello F., Sardans J. (2023). A better use of fertilizers is needed for global food security and environmental sustainability. Agric. Food Secur..

[37] Lakhdar A., Trigui M., Montemurro F. (2023). An Overview of Biostimulants’ Effects in Saline Soils. Agronomy.

[38] Sarwar B., Sher A., Ijaz M., Irfan M., Ul-Allah S. (2023). Exogenous Application of Biostimulants and Commercial Utilization. Climate-Resilient Agriculture, Vol 2: Agro-Biotechnological Advancement for Crop Production.

[39] Sun W., Shahrajabian M.H. (2023). The application of arbuscular mycorrhizal fungi as microbial biostimulant, sustainable approaches in modern agriculture. Plants.

[40] Huijbregts M.A., Steinmann Z.J., Elshout P.M., Stam G., Verones F., Vieira M., Zijp M., Hollander A., Van Zelm R. (2017). ReCiPe2016: A harmonised life cycle impact assessment method at midpoint and endpoint level. Int. J. Life Cycle Assess..

[41] Sanjuan-Delmás D., Llorach-Massana P., Nadal A., Ercilla-Montserrat M., Muñoz P., Montero J.I., Josa A., Gabarrell X., Rieradevall J. (2018). Environmental assessment of an integrated rooftop greenhouse for food production in cities. J. Clean. Prod..

[42] Romero-Gámez M., Antón A., Leyva R., Suárez-Rey E.M. (2017). Inclusion of uncertainty in the LCA comparison of different cherry tomato production scenarios. Int. J. Life Cycle Assess..

[43] Maham S.G., Rahimi A., Subramanian S., Smith D.L. (2020). The environmental impacts of organic greenhouse tomato production based on the nitrogen-fixing plant (Azolla). J. Clean. Prod..

[44] Bosona T., Gebresenbet G. (2018). Life cycle analysis of organic tomato production and supply in Sweden. J. Clean. Prod..

[45] IPPC (2006). 2006 Guidelines for National Greenhouse Gas Inventories.

[46] Djidonou D., Gao Z., Zhao X. (2013). Economic analysis of grafted tomato production in sandy soils in northern Florida. HortTechnology.

